# Supplementary material for: The causal relationship between the human gut microbiota and pyogenic arthritis: a Mendelian randomization study
Source: Front Cell Infect Microbiol. 2024 Nov 26;14:1452480. doi: 10.3389/fcimb.2024.1452480 (PMC11629706; doi:10.3389/fcimb.2024.1452480)
Supplement: Supplementary file 1 [file DataSheet1.zip › mendelian test/ebi-a-GCST90027814.csv_forest.pdf]

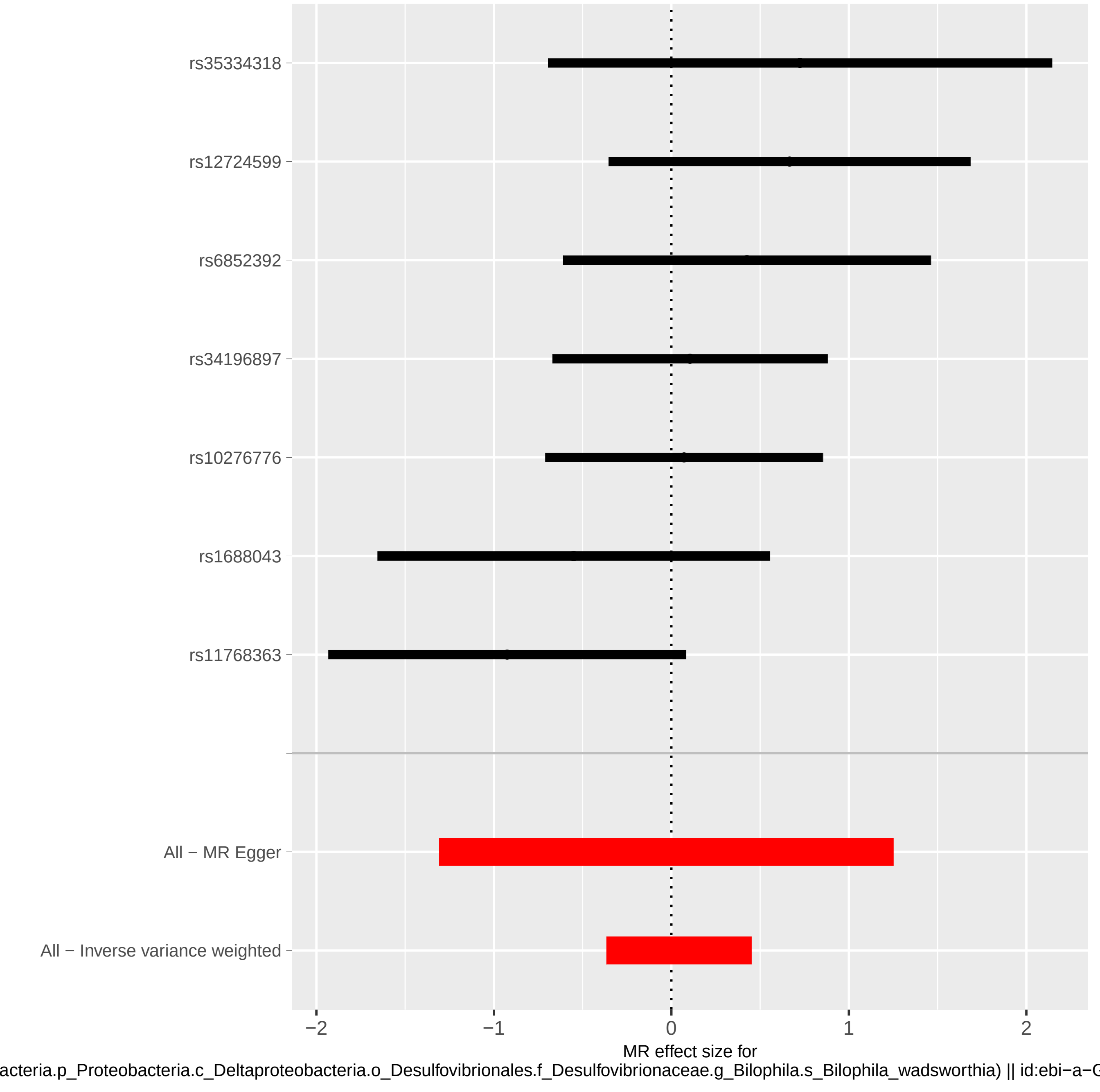

rs35334318
rs12724599
rs6852392
rs34196897
rs10276776
rs1688043
rs11768363
All − MR Egger
All − Inverse variance weighted
−2
−1
0
1
2
MR effect size for
acteria.p_Proteobacteria.c_Deltaproteobacteria.o_Desulfovibrionales.f_Desulfovibrionaceae.g_Bilophila.s_Bilophila_wadsworthia) || id:ebi−a−G
